# Cardiac Complications in Patients with Community-Acquired Pneumonia: A Systematic Review and Meta-Analysis of Observational Studies

**DOI:** 10.1371/journal.pmed.1001048

**Published:** 2011-06-28

**Authors:** Vicente F. Corrales-Medina, Kathryn N. Suh, Gregory Rose, Julio A. Chirinos, Steve Doucette, D. William Cameron, Dean A. Fergusson

**Affiliations:** 1 Department of Medicine, University of Ottawa, Ottawa, Ontario, Canada; 2 Ottawa Hospital Research Institute, Ottawa, Ontario, Canada; 3 Department of Medicine, University of Pennsylvania, Philadelphia, Pennsylvania, United States of America; Emory University, United States of America

## Abstract

**Background:**

Community-acquired pneumonia (CAP) is a leading cause of morbidity and mortality. CAP can trigger acute cardiac events. We sought to determine the incidence of major cardiac complications in CAP patients to characterize the magnitude of this problem.

**Methods and Findings:**

Two investigators searched MEDLINE, Scopus, and EMBASE for observational studies of immunocompetent adults with clinical and radiological evidence of CAP that reported any of the following: overall cardiac complications, incident heart failure, acute coronary syndromes (ACS), or incident cardiac arrhythmias occurring within 30 days of CAP diagnosis. At a minimum, studies had to establish enrolment procedures and inclusion and exclusion criteria, enrol their patients sequentially, and report the incidence of cardiac complications as a function of their entire cohorts. Studies with focus on nosocomial or health care–associated pneumonia were not included. Review of 2,176 citations yielded 25 articles that met eligibility and minimum quality criteria. Seventeen articles (68%) reported cohorts of CAP inpatients. In this group, the pooled incidence rates for overall cardiac complications (six cohorts, 2,119 patients), incident heart failure (eights cohorts, 4,215 patients), acute coronary syndromes (six cohorts, 2,657 patients), and incident cardiac arrhythmias (six cohorts, 2,596 patients), were 17.7% (confidence interval [CI] 13.9–22.2), 14.1% (9.3–20.6), 5.3% (3.2–8.6), and 4.7% (2.4–8.9), respectively. One article reported cardiac complications in CAP outpatients, four in low-risk (not severely ill) inpatients, and three in high-risk inpatients. The incidences for all outcomes except overall cardiac complications were lower in the two former groups and higher in the latter. One additional study reported on CAP outpatients and low-risk inpatients without discriminating between these groups. Twelve studies (48%) asserted the evaluation of cardiac complications in their methods but only six (24%) provided a definition for them. Only three studies, all examining ACS, carried out risk factor analysis for these events. No study analyzed the association between cardiac complications and other medical complications or their impact on other CAP outcomes.

**Conclusions:**

Major cardiac complications occur in a substantial proportion of patients with CAP. Physicians and patients need to appreciate the significance of this association for timely recognition and management of these events. Strategies aimed at preventing pneumonia (i.e., influenza and pneumococcal vaccination) in high-risk populations need to be optimized. Further research is needed to understand the mechanisms underlying this association, measure the impact of cardiac complications on other CAP outcomes, identify those patients with CAP at high risk of developing cardiac complications, and design strategies to prevent their occurrence in this population.

*Please see later in the article for the Editors' Summary*

## Abstract 2

Vicente Corrales-Medina and colleagues report estimates of the risk of cardiac complications among patients with community-acquired pneumonia from a systematic review and meta-analysis.

## Editors' Summary

**Background:**

Community-acquired pneumonia (CAP), that is, pneumonia infections contracted outside of hospital or health care settings, is a common condition and can be fatal, particularly to older people. For example, every year, an estimated 5–6 million people contract this form of pneumonia in the US, leading to 1.1 million people being admitted to hospitals and causing 60,000 deaths—the most frequent cause of infectious disease-related mortality. In the US for example, more than half of older patients who present to the hospital with CAP have preexisting chronic cardiac conditions—an important fact given that acute infections, such as CAP, can affect the cardiovascular system in various ways and precipitate acute cardiac events, such as heart failure, heart attacks, and cardiac arrhythmias.

**Why Was This Study Done?:**

Although it is bio-medically plausible that a considerable proportion of patients with CAP have cardiac complications, systematic data on the scale of this potential problem are lacking—a concerning omission given the important implications for health policy-making and direct patient care. Therefore, in this study, the researchers conducted a systematic review to examine the published literature on cardiac complications in patients with CAP in order to characterize the nature and significance of this association, and to identify areas that require further research and investigation.

**What Did the Researchers Do and Find?:**

The researchers searched MEDLINE, Scopus, and EMBASE for all relevant articles published in English, French, or Spanish languages until June 2010. The researchers used strict criteria to select appropriate articles (such as radiographic evidence of CAP) and only selected studies that had outcomes of the incidence of cardiac complications, such as incident (new or worsening) heart failure, acute coronary syndromes (acute myocardial infarction or unstable angina), and incident cardiac arrhythmias within 30 days of diagnosis of CAP.

Using these methods, the researchers identified 2,176 articles for review and selected 25 eligible papers for their analysis. Seventeen articles (68%) reported cohorts of CAP inpatients. In this group, the pooled incidence rates of overall cardiac complications (six cohorts, 2,119 patients), incident heart failure (eight cohorts, 4,215 patients), acute coronary syndromes (six cohorts, 2,657 patients), and incident cardiac arrhythmias (six cohorts, 2,596 patients), were 17.7%, 14.1%, 5.3%, and 4.7% respectively. Only three studies, (all of acute coronary syndromes) did risk factor analysis for these events. Possible risk factors identified included older age, preexisting congestive heart failure, severity of pneumonia, and the use of insulin by glucose sliding scales in hospitalized patients. No study analyzed the association between cardiac complications and other medical complications (such as acute renal failure, respiratory failure, shock, etc.) or their impact on other outcomes, such as death, in patients with CAP.

**What Do These Findings Mean?:**

Although limited to a mostly descriptive analysis, these findings clearly show that major cardiac complications occur in a significant proportion of patients with CAP, especially in those admitted to hospital. These findings have important clinical and public health implications. Clinicians should be more aware of the significance of the association between CAP and cardiac complications to better inform, treat, and manage patients. Physicians and health officials need to increase efforts to optimize the rates of influenza and pneumococcal vaccination among older people and those with chronic cardiac conditions to reduce the incidence of CAP in these high-risk populations. There needs to be more consideration given to the potential impact of cardiac complications on mortality and costs associated with CAP. Finally, these findings highlight the need for prospective, well-designed, and adequately powered studies of cardiac complications in patients with CAP. More research attention should be given to this important area in order to improve the outcomes for patients with CAP and to decrease the consequent burden on health care systems through recognition of risk, prevention, and intervention on acute cardiac complications.

**Additional Information:**

Please access these Web sites via the online version of this summary at http://dx.doi.org/10.1371/journal.pmed.1001048.

- Information can be found on Wikipedia on community-acquired pneumonia (note that Wikipedia is a free online encyclopedia that anyone can edit; available in several languages)
- The US Centers for Disease Control provide patient information on community-acquired pneumonia
- The American Heart Association provides information on heart failure, acute coronary syndromes, and arrhythmias

## Introduction

Community-acquired pneumonia (CAP) is a common and deadly condition. In the United States alone, it is estimated that each year CAP affects 5–6 million people, results in about 1.1 million hospital admissions, and causes the death of over 60,000 Americans, representing the most frequent cause of infectious disease–related mortality and, along with influenza, the overall eighth leading cause of death in this country [1],[2].

CAP occurs more frequently in the middle aged and the elderly, a population that is also at the highest risk for cardiac diseases [3],[4]. Not surprisingly, more than half of elderly patients who present to the hospital with CAP in the United States have preexisting chronic cardiac conditions, and as the population continues to age, this association will become more important [3],[5].

Acute infections, including CAP, can affect the cardiovascular system in various ways and have been recognized as precipitants of acute cardiac events [6]–[8]. Although the possibility of major cardiac complications occurring in a considerable proportion of CAP patients is very plausible, systematic data on the magnitude of this problem are remarkably scant [9]. Given the burden of CAP in North America and other western societies [1],[2], a careful characterization of the risk of cardiac complications in patients with this infection can have important implications for health policy-making and direct patient care. This systematic review examines the literature published on cardiac complications in patients with CAP in an attempt to characterize the nature and significance of this association, and to identify areas in this field that require further investigation.

## Methods

The PRISMA checklist is provided in Text S1.

### Search Strategy

Our systematic search strategy was developed to capture all articles of prognosis of CAP in which cardiac complications had been reported and is presented in Text S2. We included articles reporting in English, French, or Spanish languages. We searched the following databases: MEDLINE (from 1950 to June 13, 2010), Scopus (from 1960 to June 13, 2010), and EMBASE (from 1980 to June 13, 2010). Reference lists of selected papers were also screened for additional articles of interest.

### Outcomes

Our outcomes consisted of the incidence of cardiac complications as a combined endpoint, incident (new or worsening) heart failure, acute coronary syndromes (ACS; acute myocardial infarction or unstable angina), and incident cardiac arrhythmias within 30 d of CAP diagnosis.

### Eligibility Criteria

To ensure that the literature reviewed dealt with CAP rather than other conditions, we included only studies in which the definition of CAP was supported by radiographic evidence of acute airspace disease (new or progressing infiltrate within 48 h of presentation), and clinical signs or symptoms of pulmonary infection. Only observational studies reporting the occurrence of any of the cardiac complications of interest in their results or stating the evaluation of these outcomes in their methods were considered. At a minimum, studies had to establish enrolment procedures and inclusion and exclusion criteria in their methodological section, enrol their patients sequentially, and report the incidence of cardiac complications as a function of their entire cohorts.

We excluded studies with focus on nosocomial or health care–associated pneumonia, case series (defined as studies with ≤25 participants), articles without original data, antibiotic efficacy trials (because they are usually restricted to highly selected patients), and articles dealing primarily with pediatric patients or patients infected with the human immunodeficiency virus. We also excluded studies in which the inception time of their cohorts was beyond 48 h from the diagnosis of pneumonia.

### Selection of Studies

All titles and abstracts of the citations identified by our literature search were independently screened by two investigators (VFC-M and KNS). Relevant articles were reviewed in their entirety. Each investigator made a recommendation for inclusion or exclusion of single articles and if discordant, a third investigator solved the discrepancy (GR). When two or more articles had overlap of their populations and reported on the same cardiac outcomes, only the most inclusive article was considered.

### Data Extraction, Synthesis, and Analyses

We systematically collected data on the incidence of the cardiac complications of interest, the characteristics of the populations studied, and several aspects of the study setting and methodological design (Table S1). We contacted (by e-mail) the corresponding authors of those papers that offered no details of the methodology followed for the ascertainment of the cardiac complications of interest, and asked them to provide us with copies of the study protocols (or similar) in which this information would be available. Given the aim of our analyses, we focused on how rigorously the evaluation of medical and/or cardiac complications was established in the methodological considerations of these studies as an indicator of their risk of bias in the ascertainment and reporting of these outcomes and their quality relevant to our work. Publication bias was assessed by preparing a funnel plot for the outcome of overall cardiac complications.

Pooled incidence rates of cardiac complications were calculated separately for studies dealing with outpatients, inpatients as a whole, low-risk inpatients (e.g., inpatients with no indication for hospital admission, low-risk pneumonia severity index categories, not requiring admission to intensive care units, etc.), and high-risk inpatients (i.e., patients admitted to intensive care units). We took this approach to prevent heterogeneity in our estimates since these categories represent distinct populations of CAP patients. We performed prespecified subgroup analyses for studies of CAP inpatients by characteristics of their study setting and design, quality indicators of potential bias (see above), and attributes of their populations. Because of the limited number of studies available, these analyses are presented in a descriptive format only. Pooled event rates and their confidence intervals (CIs) were estimated using a random effects model weighted by the inverse variance. When only one study was available, the Agresti-Coull method was used. All analyses were conducted in comprehensive meta-analysis version 2.2.

## Results

**Figure 1:**
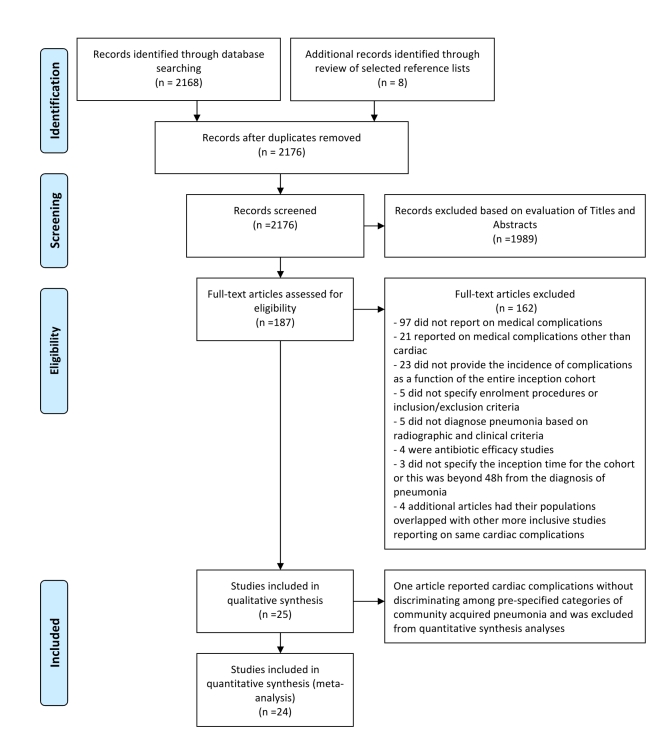
PRISMA flow diagram: selection process.

Our search strategy yielded 2,176 articles for review. A flow summary of the selection process is provided in Figure 1. Table 1 presents a summarized description of the 25 ultimately selected articles [10]–[34]. Two studies had overlapping study populations [30],[34], but since the smaller study reported on each of the four cardiac complications of interest [30], while the larger one reported only data on ACS [34], we kept both but considered only the latter for analyses related to ACS. One article reported on outpatients and inpatients as separate groups [19], and for purposes of comparative and pooled analyses, each group was treated as a distinct study. Table S1 provides the most detailed information on the setting, methodology, population, and findings of the included studies.

**Table 1:** Studies of cardiac complications in patients with CAP.

Reference	Year	*n*	Population	Design	Incidence of Cardiac Complications (%)			
					Overall Cardiac Complications^a^	Incident Heart Failure	Incident Cardiac Arrhythmias^b^	ACS^c^
Allen et al. [10]	1984	502	Inpatients	Prospective single-center	—	—	<1	—
Esposito et al. [11]	1984	38	Inpatients^d^	Prospective single-center	—	7.9	—	—
Marrie et al. [12]	1989	583	Inpatients	Prospective single-center	—	11.3	—	—
Ortqvist et al. [13]	1990	277	Inpatients	Prospective single-center	13			
Venkatesan et al. [14]	1990	73	Inpatients	Prospective single-center	—	—	11	—
Fine et al. [15]	1990	170	Outpatients and low-risk inpatients^e^	Prospective single-center	—	—	0.6	0.6
Anonymus [16]	1992	60	High-risk inpatients^f^	Retrospective multicenter	—	—	23.3	—
Leroy et al. [17]	1995	299	High-risk inpatients^f^	Retrospective single-center	2.3	—	—	—
Janssens et al. [18]	1996	99	Inpatients	Prospective single-center	—	33.3	—	—
Fine et al. [19]	1999	907	Outpatients	Prospective multicenter	—	1.4	0.9	0.1
Fine et al. [19]	1999	1,343	Inpatients	Prospective multicenter	—	20.8	9.5	3.1
Musher et al. [20]	2000	100	Inpatients^d^	Prospective single-center	—	—	—	4
Fernandez Sabé et al. [21]	2003	1,474	Inpatients	Prospective single-center	—	7.2	—	—
Fine et al. [22]	2003	608	Inpatients	Prospective multicenter	22	—	—	—
Martinez-Moragon et al. [23]	2004	91	Inpatients	Prospective single-center	5	—	—	—
Menedez et al. [24]	2004	1,424	Low-risk inpatients^e^	Prospective multicenter	—	8.7	—	—
Querol-Ribelles et al. [25]	2005	459	Low-risk inpatients^e^	Prospective single-center	—	2.6	—	—
Diaz et al. [26]	2005	113	High-risk inpatients^f^	Prospective single-center	—	24	15	—
Marrie et al. [27]	2005	586	Low-risk inpatients^e^	Prospective multicenter	—	1.4	—	0.3
McAlister et al. [28]	2005	2,471	Low-risk inpatients^e^	Prospective multicenter	5.9	—	—	—
O'meara et al. [29]	2005	582	Inpatients	Prospective multicenter	24	—	—	—
Musher et al. [30]	2007	170	Inpatients^d^	Retrospective single-center	19.4	14.7	5.9	7^g^
Becker et al. [31]	2007	391	Inpatients	Retrospective multicenter	17.4	12.3	2.8	7.9
Cabré et al. [32]	2008	117	Inpatients	Prospective single-center	—	12	4.4	0.9
Ramirez et al. [33]	2008	500	Inpatients	Retrospective single-center	—	—	—	5.8
Corrales-Medina et al. [34]	2009	206	Inpatients^d^	Retrospective single-center	—	—	—	10.7^g^

a Congestive heart failure, atrial fibrillation, severe angina or myocardial infarction or stroke [13]; acute coronary or ventricular insufficiency [17]; cardiovascular complications likely to necessitate continued hospitalization [22]; cardiac complications without further specification [23]; acute coronary syndrome and/or heart failure [28]; myocardial infarction, angina pectoris, revascularization by angioplasty/coronary artery bypass graft (CABG) or death secondary to coronary heart disease, cerebrovascular accident, congestive heart insufficiency or claudication [29]; myocardial infarction, atrial fibrillation or ventricular tachycardia or incident heart failure [30]; and myocardial infarction, atrial fibrillation, congestive heart failure or stroke [31].

b Incident atrial fibrillation [10],[14],[15],[31],[32]; cardiac dysrrhythmias/arrhythmias [16],[26]; incident atrial arrhythmia [19]; atrial flutter or fibrillation, and ventricular tachycardia, but excluding terminal arrhythmias [30].

c Myocardial infarction [15],[19],[20],[30],[31],[33]; unstable angina [27]; acute coronary syndrome [32],[34].

d Pneumococcal pneumonia [11],[20],[30]; pneumococcal and *H. influenzae* pneumonia [34].

e Inpatients without severe vital signs or metabolic abnormalities, altered mental status, suppurative complications or coexisting medical conditions requiring hospitalization [15]; inpatients who survived the first 48 h of hospitalization [24], inpatients not initially admitted to the intensive care unit [25],[28]; inpatients with pneumonia severity index (PSI) risk classes I–II [27].

f Inpatients admitted to the intensive care unit (ICU).

g For ACS, patients from Musher et al. (2007) [30] were included in Corrales-Medina et al. (2009) [34].

### Assessment of Quality and Risk of Bias

The evaluation of medical and cardiac complications was documented in the methodological considerations of 68% (17 cohorts, *n* = 12,068) and 48% (12 cohorts, *n* = 9,344) of the 25 studies, respectively; whereas definitions of the cardiac complications of interest were available for 24% (six cohorts, *n* = 4,125) of them (Table S1). Table 2 provides the stratification of the articles by these quality elements for each of the cardiac complications of interest. The definitions used for the ascertainment of cardiac complications in the studies for which this information was available is provided in Table S2. Due to the low number of studies, a visual inspection of our funnel plot for overall cardiac complications did not allow for a meaningful assessment of publication bias (Figure S1).

**Table 2:** Studies of cardiac complications in patients with CAP: Methodological considerations for the ascertainment of medical and cardiac complications.

Outcome	Total *References* (*n*)	Methods Section Established the Following:		
		Evaluation of Medical Complications *References* (*n*)	Evaluation of Cardiac Complications *References* (*n*)	Definition for Cardiac Complications *References* (*n*)
**Overall cardiac complications**				
Outpatients	—	—	—	—
Inpatients	*[13],[22],[23],[29]–[31]* (2,119)	*[13],[22],[23],[29]–[31]* (2,119)	*[22],[29]–[31]* (1,751)	*[22],[30],[31]* (1,169)
Low-risk inpatients	*[28]* (2,471)	*[28]* (2,471)	*[28]* (2,471)	—
High-risk inpatients	*[17]* (299)	*[17]* (299)	—	—
**Incident heart failure**				
Outpatients	*[19]* (907)	*[19]* (907)	*[19]* (907)	*[19]* (907)
Inpatients	*[11],[12],[18],[19],[21],[30]–[32]* (4,215)	*[12],[19],[21],[30],[31]* (3,054)	*[19],[30],[31]* (1,904)	*[19],[30],[31]* (1,904)
Low-risk inpatients	*[24],[25],[27]* (2,469)	*[24],[25]* (1,883)	*[24],[25]* (1,883)	—
High-risk inpatients	*[26]* (113)	*[26] (113)*	*[26]* (113)	—
**ACS**				
Outpatients	*[19]* (907)	*[19]* (907)	*[19] (907)*	*[19]* (907)
Inpatients	*[19],[20],[31]–[33],[24]* (2,657)	*[19],[31],[33],[34]* (2,440)	*[19],[31],[33],[34]* (2,440)	*[19],[31],[33],[34]* (2,440)
Low-risk inpatients	*[27]* (586)	—	—	—
High-risk inpatients	—	—	—	—
**Incident cardiac arrhythmias**				
Outpatients	*[19]* (907)	*[19]* (907)	*[19]* (907)	*[19]* (907)
Inpatients	*[10],[14],[19],[30]–[32]* (2,596)	*[19],[30],[31] (1,904)*	*[19],[30],[31]* (1,904)	*[19],[30],[31]* (1,904)
Low-risk inpatients	—	—	—	—
High-risk inpatients	*[16],[26]* (173)	*[26] (113)*	*[26]* (113)	—

One study reporting the incidence of ACS and incident cardiac arrhythmias on outpatients and low-risk inpatients without making distinction between them was not included in this table [15].

### Overall Cardiac Complications

**Figure 2:**
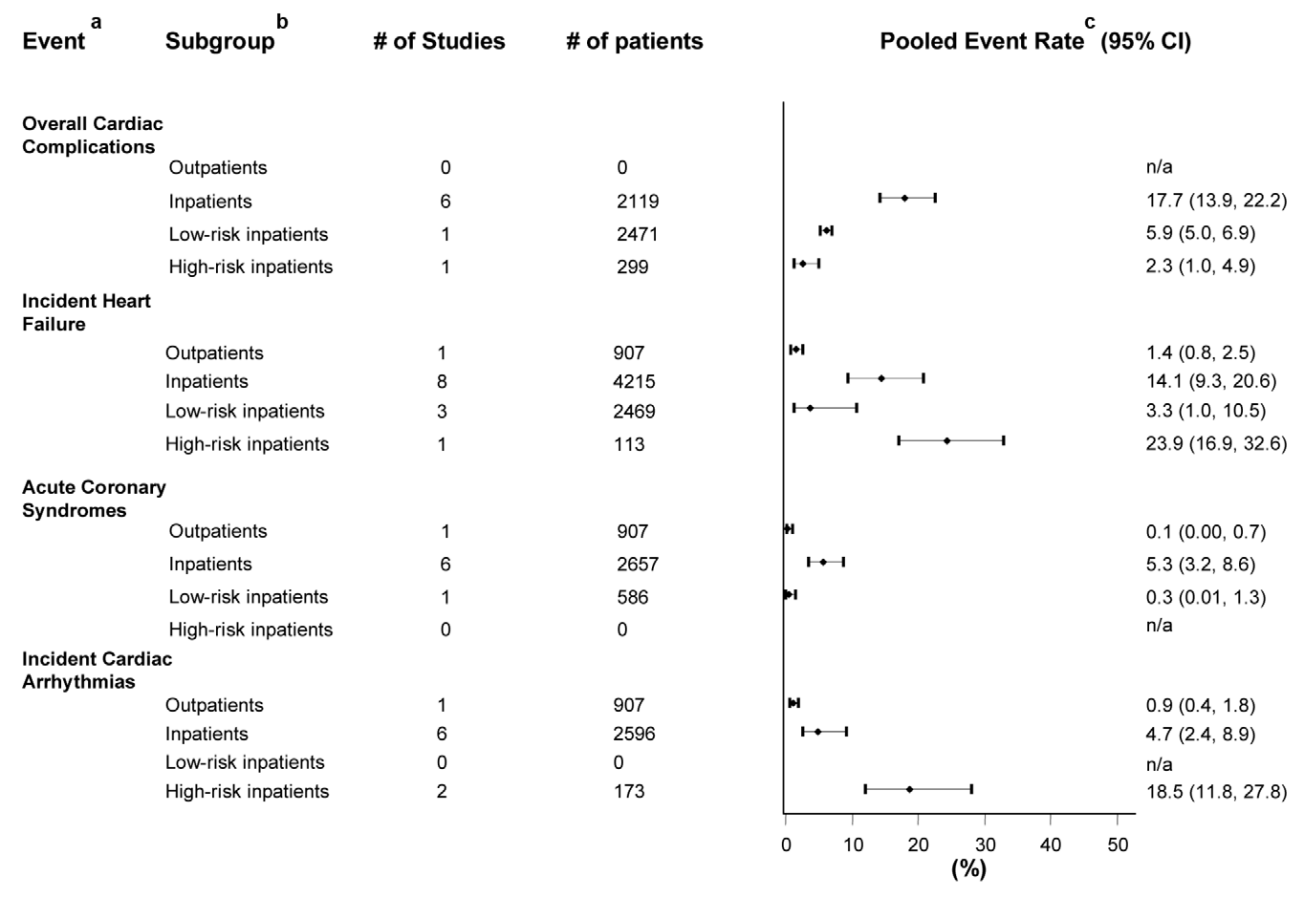
Pooled rates of the incidence of cardiac complications in patients with CAP. One study reporting the incidence of ACS and incident cardiac arrhythmias on outpatients and low-risk inpatients without making distinction between them was not included in the calculations of pooled rates [15]. ^a^For definitions refer to Table 1. ^b^Low-risk inpatients included studies of inpatients with no indication for hospital admission, pneumonia severity index (PSI) risk-classes I–II, not initially admitted to intensive care units or who survived the first 48 h of hospitalization. High-risk inpatients included patients admitted to intensive care units. ^c^When only one study was available, the reported rate represents the study event rate.

Six studies focused on CAP inpatients [13],[22],[23],[29]–[31], one on low-risk inpatients [28], and one on high-risk inpatients [17]; with pooled incidence rates of 17.7% (CI 13.9–22.2) 5.9% (5.0–6.9), and 2.3% (1.0–4.9) for each of these groups, respectively (Figure 2).

### Incident Heart Failure

One study reported on outpatients [19], eight on inpatients [11],[12],[18],[19],[21],[30]–[32], three on low-risk inpatients [24],[25],[27] and one on high-risk inpatients [26]. The pooled incidence rates for each of these groups were 1.4% (0.8–2.5), 14.1% (9.3–20.6), 3.3% (1.0–10.5), and 23.9% (16.9–32.6), respectively (Figure 2).

### Acute Coronary Syndromes

Five studies reported only on myocardial infarction [15],[19],[20],[31],[33], one on unstable angina [27], and two on ACS in general [32],[34]. One study reported on outpatients and low-risk inpatients without making distinction between them and was not accounted for in the calculations of pooled rates [15]. One study focused on outpatients [19], six on inpatients [19],[20],[31]–[34], and one on low-risk inpatients [27], with pooled incidence rates of 0.1% (0.0–0.7), 5.3% (3.2–8.6), and 0.3% (0.01–1.3), respectively (Figure 2).

### Incident Cardiac Arrhythmias

Five studies reported solely on atrial fibrillation [10],[14],[15],[31],[32], one study on all atrial arrhythmias [19], two on cardiac arrhythmias in general [16],[26], and one on atrial fibrillation (88% of the cases) and ventricular arrhythmias (excluding terminal arrhythmias) [30]. One study reporting on outpatients and low-risk inpatients without making distinction between them was not included in the calculations of pooled rates [15]. One study evaluated outpatients [19], six studied inpatients [10],[14],[19],[30]–[32], and two focused on high-risk inpatients [16],[26]; giving pooled incidence rates of 0.9% (0.4–1.8), 4.7% (2.4–8.9), and 18.5% (11.8–27.8), respectively (Figure 2).

### Subgroup Analyses

Tables 3 and 4 describe the results of prespecified subgroup analyses of studies on CAP inpatients by characteristics of their setting, methodology, and population. Incident cardiac arrhythmias and incident heart failure were more common in papers published before 2000 and those coming from Europe. Overall cardiac complications and ACS, on the other hand, were more frequent in studies published between 2000 and 2005, and after 2005, respectively, and in papers coming from North America. All cardiac complications but ACS were more common in studies of prospective multicenter design. Additionally, all cardiac complications tended to occur more frequently in studies in which the evaluation of these events was stated in their methodological considerations or a definition for their ascertainment was available.

**Table 3:** Studies of cardiac complications in inpatients with CAP: Subgroup analysis by study setting and design.

Subgroups	Overall Cardiac Complications		Incident Heart Failure		ACS		Incident Cardiac Arrhythmias	
	*n* Studies	Incidence (95% CI)	*n* Studies	Incidence (95% CI)	*n* Studies	Incidence	*n* Studies	Incidence (95% CI)
Year:								
Before 2000	1	13.0 (9.5–17.5)	4	18.1 (10.8–28.7)	1	3.1 (2.3–4.2)	3	5.1 (1.5–15.7)
2000–2005	3	19.0 (13.3–26.3)	1	7.2 (6.0–8.6)	1	4.0 (1.2–10.2)	—	—
2006+	2	18.0 (15.1–21.4)	3	12.9 (10.5–15.6)	4	7.1 (4.5–10.8)	3	4.1 (2.5–6.5)
Region:								
North America	4	21.0 (18.2–24.2)	5	13.9 (9.7–19.5)	5	5.9 (3.6–9.5)	3	5.6 (2.6–11.8)
Latin America	—	—	—	—	—	—	—	—
Europe	2	9.3 (4.0–20.2)	3	15.1 (4.7–39.0)	1	0.9 (0.0–5.2)	2	7.1 (2.8–17.10
Asia	—	—	—	—	—	—	—	—
Africa	—	—	—	—	—	—	1	1.0 (0.4–2.4)
Oceania	—	—	—	—	—	—	—	—
Design:								
Prospective single—center	2	9.3 (4.0–20.2)	5	13.0 (6.9–23.0)	2	2.3 (0.5–9.5)	3	3.7 (0.9–14.2)
Prospective multicenter	2	23.0 (20.7–25.5)	1	20.8 (18.7–23.0)	1	3.1 (2.3–4.2)	1	9.5 (8.1–11.2)
Retrospective	2	18.0 (15.1–21.4)	2	13.1 (10.5–16.1)	3	7.8 (5.6–10.8)	2	4.0 (1.9–8,2)
Evaluation of medical complications stated in Methods section:								
No	—	—	3	16.7 (5.9–39.0)	2	2.3 (0.5–9.5)	3	3.7 (0.9–14.2)
Yes	6	17.7 (13.9–22.2)	5	12.6 (7.8–19.8)	4	6.2 (3.6–10.5)	3	5.6 (2.6–11.8)
Evaluation of cardiac complications stated in Methods section:								
No	2	9.3 (4.0–20.2)	5	13.0 (6.9–23.0)	2	2.3 (0.5–9.5)	3	3.7 (0.9–14.2)
Yes	4	21.0 (18.2–24.2)	3	15.9 (10.8–22.8)	4	6.2 (3.6–10.5)	3	5.6 (2.6–11.8)
Definition of cardiac complications provided in Methods section:								
No	3	13.5 (6.7–25.3)	5	13.0 (6.9–23.0)	2	2.3 (0.5–9.5)	3	3.7 (0.9–14.2)
Yes	2	20.0 (17.0–23.2)	3	15.9 (10.8–22.8)	4	6.2 (3.6–10.5)	3	5.6 (2.6–11.8)

One study reporting the incidence of ACS and incident cardiac arrhythmias on outpatients and low-risk inpatients without making distinction between them was not included in this table [15].

**Table 4:** Studies of cardiac complications in inpatients with CAP: subgroup analysis by baseline characteristics of the population.

Subgroups	Overall Cardiac Complications		Incident Heart Failure		ACS		Incident Cardiac Arrhythmias	
	*n* Studies	Incidence (95% CI)	*n* Studies	Incidence (95% CI)	*n* Studies	Incidence (95% CI)	*n* Studies	Incidence (95% CI)
Age								
<50	—	—	—	—	—	—	1	1.0 (0.4–2.4)
50–65	—	—	3	13.9 (7.8–23.5)	2	3.2 (2.4–4.2)	1	9.5 (8.1–11.2)
>65	5	17.2 (13.0–22.4)	4	14.3 (6.9–27.4)	4	7.1 (4.5–10.8)	3	5.1 (2.1–11.7)
Not reported	1	19.4 (14.1–26.0)	1	14.7 (10.1–20.9)	—	—	1	5.9 (3.1–10.6)
Gender:								
<50% Male	4	16.7 (11.6–23.5)	2	19.1 (3.7–59.0)	—	—	—	—
>50%+ Male	2	18.0 (15.1–21.4)	6	12.5 (8.1–18.8)	6	5.3 (3.2–8.6)	6	4.7 (2.4–8.9)
Preexisting cardiac/cardiovascular disease:								
<25%	—	—	—	—	—	—	—	—
25–50%	—	—	—	—	—	—	—	—
50%+	—	—	—	—	—	—	—	—
Not reported	6	17.7 (13.9–22.2)	8	14.1 (9.3–20.6)	6	5.3 (3.2–8.6)	6	4.7 (2.4–8.9)
Coronary artery disease:								
<25%	—	—	1	12.0 (7.1–19.2)	1	0.9 (0.0–5.2)	1	4.3 (1.6–9.9)
25–50%	2	23.0 (20.7–25.5)	3	13.9 (7.8–23.5)	3	5.8 (2.8–11.3)	1	9.5 (8.1–11.2)
50%+	—	—	—	—	—	—	—	—
Not reported	4	14.5 (10.4–19.8)	4	15.0 (7.5–27.9)	2	6.5 (3.5–11.7)	4	3.8 (1.5–9.2)
Congestive heart failure:								
<25%	4	18.8 (14.3–24.3)	2	16.3 (9.5–26.5)	3	6.4 (3.0–13.3)	2	5.4 (1.6–16.8)
25–50%	—	—	1	12.0 (7.1–19.2)	3	4.1 (1.9–8.5)	1	4.3 (1.6–9.9)
50%+	—	—	—	—	—	—	—	—
Not reported	2	15.9 (10.6–23.1)	5	13.5 (7.4–23.5)	—	—	3	4.2 (1.1–14.1)
Diabetes mellitus:								
<25%	3	21.3 (17.9–25.1)	5	11.6 (6.8–19.0)	3	6.4 (3.0–13.3)	2	5.4 (1.6–16.8)
25–50%	1	5.5 (2.1–12.5)	1	12.0 (7.1–19.2)	2	2.9 (0.5–16.0)	1	4.3 (1.6–9.9)
50%+	—	—	—	—	—	—	—	—
Not reported	2	15.6 (10.6–23.1)	2	23.5 (9.0–48.8)	1	4.0 (1.2–10.2)	3	4.2 (1.1–14.1)
Chronic obstructive pulmonary disease:								
<25%	4	15.2 (10.0–22.4)	3	9.1 (5.8–14.2)	1	7.9 (5.6–11.1)	1	2.8 (1.5–5.0)
25–50%	1	22.0 (18.9–25.5)	2	15.6 (8.4–27.2)	3	5.8 (2.8–11.3)	1	9.5 (8.1–11.2)
50%+	—	—	1	12.0 (7.1–19.2)	2	2.3 (0.5–9.5)	1	4.3 (1.6–9.9)
Not reported	1	19.4 (14.1–26,0)	2	23.5 (9.0–48.8)	—	—	3	4.2 (1.1–14.1)
Smoking:								
<25%	1	24.1 (20.8–27.7)	—	—	—	—	—	—
25–50%	—	—	2	11.1 (8.9–13.9)	1	5.8 (4.0–8.2)	1	11.0 (5.4–20.4)
50%+	—	—	—	—	2	7.2 (2.7–17.7)	—	—
Not reported	5	16.2 (12.2–21.1)	6	15.4 (9.3–24.3)	3	3.9 (1.6–9.2)	5	3.9 (1.8–8.4)

One study reporting the incidence of ACS and incident cardiac arrhythmias on outpatients and low-risk inpatients without making distinction between them was not included in this table [15].

Incident heart failure, incident cardiac arrhythmias, and ACS tended to be more common in studies with older populations and higher rates of preexisting coronary artery disease, but not in those with higher prevalence of preexisting congestive heart failure. Studies of predominantly female populations had higher incidences of incident heart failure, whereas the opposite was observed for overall cardiac complications. Rates of all cardiac complications but incident heart failure were lower in studies of patients with higher prevalence of diabetes mellitus. While overall cardiac complications, incident heart failure and incident cardiac arrhythmias were more common in studies with higher prevalence of chronic obstructive pulmonary disease, the opposite was observed for ACS. Finally, ACS occurred more commonly in studies of patients with higher rates of smoking.

### Risk Factors and Impact of Cardiac Complications on CAP Outcomes

Only three studies [31],[33],[34], all dealing with CAP and ACS, attempted to analyze risk factors for the occurrence of cardiac complications. Possible risk factors identified included older age, preexisting congestive heart failure [34], severity of pneumonia [33], and the use of insulin by glucose sliding scales in hospitalized patients [31]. No study analyzed the association of cardiac complications with the development of other medical complications (i.e., acute renal failure, respiratory failure, shock, etc.), or the impact of these events on other CAP outcomes (i.e., mortality).

## Discussion

Our main finding is that major cardiac complications occur in a significant proportion of patients with CAP, especially in those requiring hospitalization for this infection. The pooled incidence rates of overall cardiac complications, incident heart failure, ACS, and incident arrhythmias in hospitalized patients with CAP were 17.7%, 14.1%, 5.3%, and 4.7%, respectively. Given the burden of CAP in North America and other western societies [1],[2], these pooled findings have important implications. Firstly, clinicians need to realize the significance of this association for appropriate clinical alertness and to better inform CAP patients about the risk of cardiac complications once the diagnosis of pneumonia is made. Secondly, physicians and health officials need to increase efforts to optimize the rates of influenza and pneumococcal vaccination among the elderly and those with chronic cardiac conditions to reduce the incidence of CAP in these high-risk populations. Thirdly, attention needs to be directed to the potential impact of cardiac complications in the mortality and cost associated with CAP. Finally, the research community needs to urgently direct more efforts to the study of this area.

Our results expand on the findings of Fine et al. [9], who in the only previous systematic review on this topic reported four CAP cohorts (232 patients total) with a pooled incidence of heart failure of 8.6%. Our study not only confirms that incident heart failure is common in the course of CAP but suggests that its occurrence in patients hospitalized with this infection may be much higher than previously realized, and that ACS and cardiac arrhythmias are also remarkably frequent in this population.

Incident heart failure can be precipitated by CAP by several mechanisms [7],[30]. Acute inflammation can not only depress myocardial function, as it is well described in septic states [35],[36], but it can also increase large artery stiffness and the pulse wave reflections from peripheral middle-sized and small arteries that return to the heart in late systole, increasing left ventricular afterload and raising oxygen consumption [37]. Hypoxemia associated with CAP can raise pulmonary arterial pressure and right ventricular afterload while impairing myocardial oxygen delivery [7]. Tachycardia, common in acute infections, increases myocardial oxygen needs but shortens the diastolic period in which coronary perfusion occurs [38]–[40]. The net result of these effects is a shift in the metabolic supply/demand ratio of the myocardium and further impairment of its function. These changes are presumed to be of greater significance in patients with preexisting cardiac disease. In addition, incident heart failure in CAP can result from myocardial inflammation (myocarditis), a complication well described in patients with pneumonia mainly of viral origin, and that could have been underrepresented in previous investigations because of a lack of adequate noninvasive techniques for its identification (i.e., cardiac magnetic resonance imaging) [41]. Finally, we realize that acute renal impairment, common in hospitalized CAP patients [19], can also play a role in this setting [42].

Acute infections, including CAP, can also trigger the occurrence of ACS, and clinical studies have shown a significant temporal increase in the risk of ACS soon after the development of respiratory infections [34],[43],[44]. Surges of biomechanical stress, as a result of increased sympathetic activity and other hemodynamic changes (i.e. alterations of the circulatory volume and the systemic and coronary vascular tone), can prompt plaque rupture [7]. Acute infections can also promote plaque disruption by increasing intraplaque inflammatory activity [7]. In this setting, thrombus formation over a disrupted coronary plaque—a key step in the development of ACS—would be favoured by infection-induced prothrombotic changes in the blood and endothelium [7]. In addition, preexisting coronary artery disease that doesn't produce myocardial ischemia under baseline conditions can result in significant ischemia in the face of increased metabolic demands associated with CAP (i.e., demand ischemia; see above).

Most of the cardiac arrhythmias reported in the reviewed studies represented atrial tachyarrhythmias, particularly atrial fibrillation. Abnormalities in the cardiac conduction system in the setting of acute pneumonia have been recognized since the early 20th century and consistently confirmed thereafter [45],[46]. More recently, a study of more than 800,000 patients admitted to the hospital with atrial fibrillation as a secondary condition found that in patients 65 y of age or older, the second leading primary diagnosis was pneumonia (7%), after only congestive heart failure (13%), and before acute myocardial infarction (6%) [8].

While incident heart failure, ACS, and cardiac arrhythmias constitute distinct clinical entities, they share many pathophysiological bases and risk factors, and the occurrence of one of them can as well trigger the development of the others. While only one study in our review clearly documented the frequent concomitant occurrence of more than one cardiac complication in CAP patients [30], we think that this scenario is likely to be rather common.

Our study highlights several shortcomings of the medical literature on this area, which might influence our interpretations. Only a small proportion of studies primarily focused on cardiac outcomes and very few provided a definition for them, raising concerns for potential bias in the ascertainment and/or reporting of these events in those studies that did not. This is relevant especially for incident heart failure and ACS since their manifestations can overlap with those of CAP and other associated conditions (i.e., lung injury). Nevertheless, the few studies that provided clear definitions for the cardiac complications of interest consistently reported substantial incidences of these outcomes, providing reassurance for the validity of our findings. Only three studies of CAP and ACS looked at possible risk factors for the occurrence of these events but their analyses were largely underpowered and limited [31],[33],[34]. While it seems intuitive to think that the presence of preexisting cardiac conditions should have an important effect on the risk of cardiac complications in patients with CAP, further research will be needed to delineate their significance in this setting. As well, the association of cardiac complications with the development of other medical complications (i.e., acute renal failure, respiratory failure, shock, etc.), or the impact of these events in other CAP outcomes (i.e., mortality) is yet to be established. Finally, little is known about the timing of these complications, and only one study suggested that ACS in CAP patients tend to occur within few days after hospital admission [34].

Our work has limitations beyond the methodological shortcomings of the individual analyzed studies. We cannot rule out potential publication bias against studies that found no significant occurrences of cardiac complications. Additionally, we can only assume that the diagnostic evaluations in individual studies were performed in a uniform manner and the ascertainment of cardiac events was correct. The small number of studies dealing with CAP populations other than inpatients as a whole prevents us from drawing firm conclusions on the incidence of cardiac complications in these groups; and the finding of a counter-intuitively lower incidence of overall cardiac complications in high-risk CAP inpatients could be explained by this factor. The implied differences in the management of inpatients and outpatients may have led to the under-reporting of cardiac events in studies dealing with the latter group. Although studies of cardiac complications in CAP inpatients were the most common in our review, their number was still underpowered for performing adequate analyses (i.e., meta-regression) of factors that could account for the heterogeneity in their findings, and any appreciable difference observed in our subgroup analyses should be viewed in this context. Our review was limited to cardiac complications occurring within 30 d from presentation with CAP because it is assumed that the influence of the infection on patients' comorbid conditions is maximal during this time [47]; however, it is plausible that this influence can go beyond this period as it has been suggested by recent studies [44],[48]. Likewise, many of the mechanisms implicated in the development of cardiac complications in patients with CAP could account for similar occurrences in other infectious and noninfectious acute inflammatory states [7]. In fact, evidence suggests that acute infections of the urinary and gastrointestinal tract are also associated with increased risk of ACS in the short term [7],[44],[49]. However, exploring the magnitude of these associations was beyond the scope of our review.

Our findings highlight the urgent need for prospective, well-designed, and adequately powered studies of cardiac complications in patients with CAP. Investigations should focus on identifying risk factors for the occurrence of cardiac complications in this population and developing strategies to identify those CAP patients at high risk for developing these events. These strategies may include clinical scoring systems, biomarker-based approaches, noninvasive cardiac imaging, or a combination of these. Studies are also needed to characterize the impact of cardiac complications on the mortality and resource utilization associated with CAP. Careful mechanistic studies of the pathophysiology of cardiac complications in the course of CAP and the role of preexisting heart disease in their development should serve for the appropriate design of interventions aimed at preventing their occurrence in high-risk groups. As an example, discriminating between acute plaque rupture versus demand ischemia as the factor driving the occurrence of ACS in this population will have important and obvious therapeutic implications. Such interventions will need to be tested in randomized clinical trials. The ultimate goal will be to improve the outcomes of patients with CAP and to decrease the burden that this disease imposes on our health care systems through recognition of risk, prevention, and intervention on acute cardiac complications.

## Supporting Information

Figure S1 Funnel plot for studies of CAP that reported the incidence of overall cardiac complications.
(TIF)

Table S1 Details of the setting, design, and population of studies of cardiac complications in patients with CAP.
(DOC)

Table S2 Definitions of cardiac complications used in studies of CAP.
(DOC)

Text S1 PRISMA checklist.
(DOC)

Text S2 Search strategy.
(DOC)

## Abbreviations

ACS: acute coronary syndromes
CAP: community-acquired pneumonia
