# Supplementary figures and images for: Cardiac Complications in Patients with Community-Acquired Pneumonia: A Systematic Review and Meta-Analysis of Observational Studies
Source: PLoS Med. 2011 Jun 28;8(6):e1001048. doi: 10.1371/journal.pmed.1001048 (PMC3125176; doi:10.1371/journal.pmed.1001048)

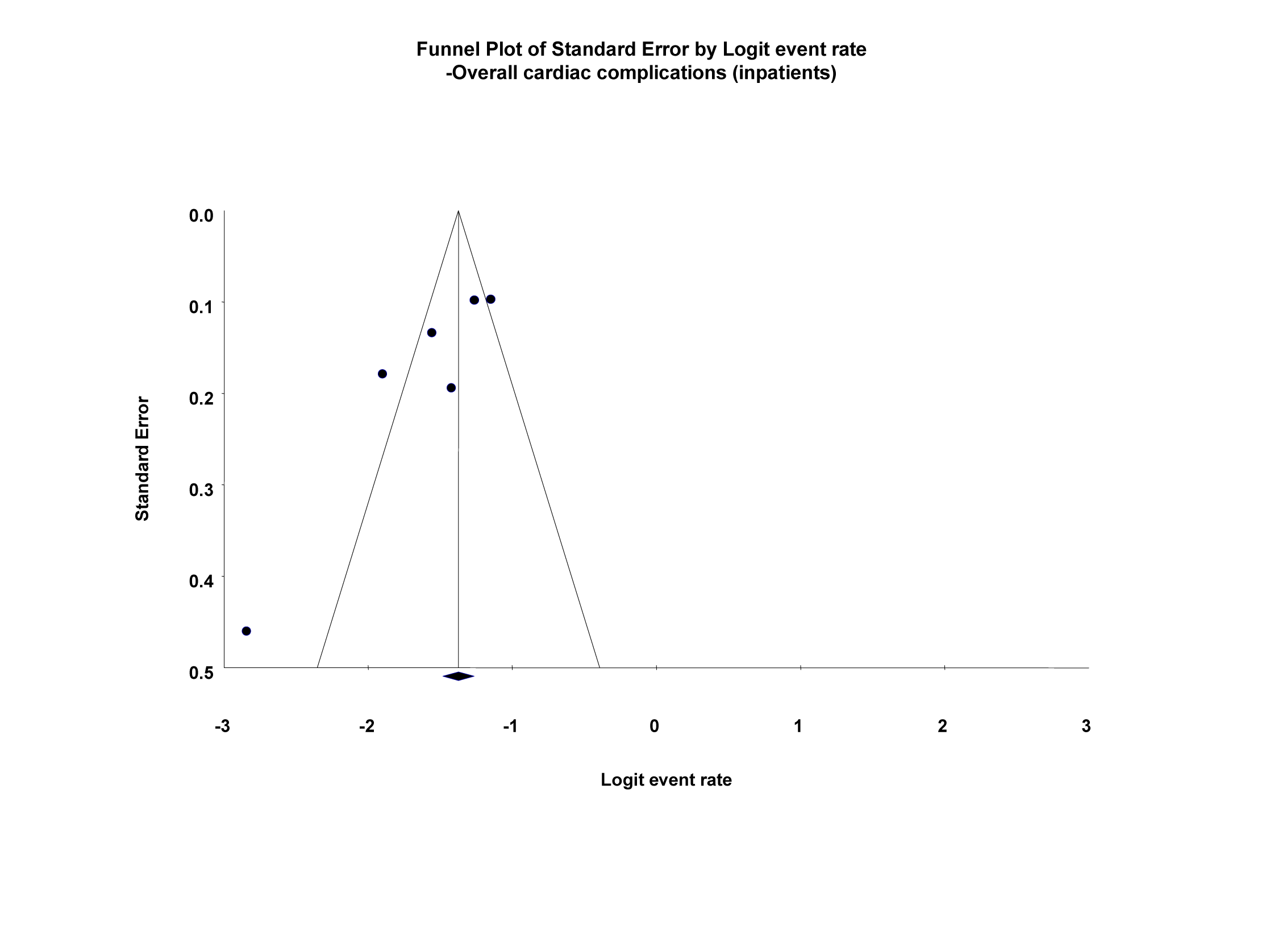

Supplement: Figure S1 — Funnel plot for studies of CAP that reported the incidence of overall cardiac complications. (TIF) [file pmed.1001048.s001.tif]
