# Supplementary material for: Cardiac Complications in Patients with Community-Acquired Pneumonia: A Systematic Review and Meta-Analysis of Observational Studies
Source: PLoS Med. 2011 Jun 28;8(6):e1001048. doi: 10.1371/journal.pmed.1001048 (PMC3125176; doi:10.1371/journal.pmed.1001048)
Supplement: Table S1 — Details of the setting, design, and population of studies of cardiac complications in patients with CAP. (DOC) [file pmed.1001048.s002.doc]

| **Reference** | **Year** | **n** | **Population** | **Country** | **Design** | **The Methods section stated the following:** | | | **Mean age** | **Male gender (%)** | **Prevalence of comorbidities at presentation with CAP** | | | | | | **Mortality**  **(%)** | **Incidence of cardiac complications**  **(%)** | | | |
| --- | --- | --- | --- | --- | --- | --- | --- | --- | --- | --- | --- | --- | --- | --- | --- | --- | --- | --- | --- | --- | --- |
|  |  |  |  |  |  | Evaluation of medical complications | Evaluation of cardiac complications | Definition of cardiac complications |  |  | Cardiovascular disease | Congestive heart failure | Coronary artery disease | Diabetes | Chronic obstructive pulmonary disease | Smoking |  | Overall cardiac compilations ^a^ | Incident heart failure | Incident cardiac arrhythmias ^b^ | Acute coronary syndromes ^c^ |
| Allen et al [10] | 1984 | 502 | Inpatients | Zambia | Prospective single-center | No | No | No | 39 | 72.1 | - | - | - | - | - | - | 5.6  (in-hospital) | - | - | <1 | - |
| Esposito et al [11] | 1984 | 38 | Inpatients ^d^ | US | Prospective single-center | No | No | No | 61 | 39.5 | - | - | 28.9 | 13.2 | 18.4 | 39.5 | 13.2  (in-hospital) | - | 7.9 | - | - |
| Marrie et al[12] | 1989 | 583 | Inpatients | Canada | Prospective single-center | Yes | No | No | 60 | 64.7 | - | - | 25 | 11.8 | 32 | 39 | 21  (in-hospital) | - | 11.3 | - | - |
| Ortqvist et al [13] | 1990 | 277 | Inpatients | Sweden | Prospective single-center | Yes | No | No | 62 | 43 | - | - | - | - | 9.4 | - | 4  (in-hospital) | 13 |  |  |  |
| Venkatesan et al [14] | 1990 | 73 | Inpatients | UK | Prospective single-center | No | No | No | 79 ^e^ | 52.1 | - | - | - | - | - | 27.4 | 21.9  (14-day) | - | - | 11 | - |
| Fine et al [15] | 1990 | 170 | Outpatients (94) and low-risk inpatients (76) ^f^ | US | Prospective single-center | Yes | Yes | No | 52.4 | 56 | - | 6 | 15 | 10 | 6 | 32 | 3.5  (6-week) | - | - | 0.6 | 0.6 |
| Anonymus [16] | 1992 | 60 | High-risk inpatients ^g^ | UK | Retrospective  multicenter | No | No | No | 54 | 57 | 15 | - | - | 7 | 32 | 72 | 48  (in-hospital) | - | - | 23.3 | - |
| Leroy et al [17] | 1995 | 299 | High-risk inpatients ^g^ | France | Retrospective  Single-center | Yes | No | No | 63.9 | 62.9 | - | - | - | - | - | - | 28.4  (ICU) | 2.3 | - | - | _ |
| Janssens et al [18] | 1996 | 99 | Inpatients | Switzerland | Prospective single-center | No | No | No | 85 | 36.4 | - | - | - | - | - | - | 14  (30-day) | _ | 33.3 | _ | _ |
| Fine et al [19] | 1999 | 907 | Outpatients | US and Canada | Prospective multicenter | Yes | Yes | Yes | 44.7 | 46.7 | - | 3 | 6 | 3.9 | 14.3 | - | 0.6  (30-day) | _ | 1.4 | 0.9 | 0.1 |
| Fine et al [19] | 1999 | 1343 | Inpatients | US and Canada | Prospective multicenter | Yes | Yes | Yes | 64.3 | 52.3 | - | 16.8 | 26 | 14.7 | 33.9 | - | 8  (30-day) | _ | 20.8 | 9.5 | 3.1 |
| Musher et al [20] | 2000 | 100 | Inpatients ^d^ | US | Prospective single-center | No | No | No | 61.5 | 98 | - | 22 | - | 12 | 50 | 68 | 17  (30-day) | _ | _ | _ | 4 |
| Fernandez Sabé et al [21] | 2003 | 1474 | Inpatients | Spain | Prospective single-center | Yes | No | No | 65.2 | 69.9 | 22.2 | - | - | 16.9 | 24.4 | - | 8.2  (30-day) | _ | 7.2 | _ | _ |
| Fine et al [22] | 2003 | 608 | Inpatients | USA | Prospective multicenter | Yes | Yes | Yes | 68.5 | 45.5 | - | 23 | 38 | 23.2 | 48 | - | 8.5  (30-day) | 22 | _ | _ | _ |
| Martinez-Moragon et al [23] | 2004 | 91 | Inpatients | Spain | Prospective single-center | Yes | No | No | 75.4 | 44 | - | 15.4 | NR | 26.4 | 20.1 | - | 10.9  (in-hospital) | 5 | _ | _ | _ |
| Menedez et al [24] | 2004 | 1424 | Low-risk inpatients ^f^ | Spain | Prospective multicenter | Yes | Yes | No | 68 | 66.9 | 30.1 | - | - | 18.2 | 22.8 | 24.6 | 5.6  (30-day) | _ | 8.7 | _ | _ |
| Querol-Ribelles et al [25] | 2005 | 459 | Low-risk inpatients ^f^ | Spain | Prospective single-center | Yes | Yes | No | 70.5 | 70.8 | - | 17.2 | 13.1 | 25.1 | 41.2 | 17.9 | 8.7  (30-day) | _ | 2.6 | _ | _ |
| Diaz et al [26] | 2005 | 113 | High-risk inpatients  ^g^ | Chile | Prospective single-center | Yes | Yes | No | 73 | 58.4 | 44 | - | - | 23 | 30 | 35 | 16.8  (30-day) | _ | 24 | 15 | _ |
| Marrie et al [27] | 2005 | 586 | Low-risk inpatients  ^f^ | Canada | Prospective multicenter | No | No | No | 46.5 | 41 | - | - | - | - | - | - | 0.9  (30-day) | _ | 1.4 | _ | 0.3 |
| McAlister et al [28] | 2005 | 2471 | Low-risk inpatients ^f^ | Canada | Prospective multicenter | Yes | Yes | No | 75 | 52 | - | 18 | - | 16 | - | - | 9  (in-hospital) | 5.9 | _ | _ | _ |
| O’meara et al [29] | 2005 | 582 | Inpatients | United States | Prospective multicenter | Yes | Yes | No | 75 | 49.3 | - | 9 | 30 | 18 | 20 | 17 | 10.3  (in-hospital) | 24 | _ | _ | _ |
| Musher et al [30] | 2007 | 170 | Inpatients ^d^ | US | Retrospective single-center | Yes | Yes | Yes | NR | >95 | - | - | - | - | - | - | 12.4  (in-hospital) | 19.4 | 14.7 | 5.9 | 7 ^h^ |
| Becker et al [31] | 2007 | 391 | Inpatients | Canada | Retrospective  multicenter | Yes | Yes | Yes | 76.6 | 50.4 | - | 17.6 | - | 23 | 21.5 | - | 10.9  (in-hospital) | 17.4 | 12.3 | 2.8 | 7.9 |
| Cabré et al [32] | 2008 | 117 | Inpatients | Spain | Prospective single-center | No | No | No | 84.7 | 59 | - | 25.6 | 15.4 | 31.6 | 56.4 | - | 12.8  (30-day) | _ | 12 | 4.4 | 0.9 |
| Ramirez et al [33] | 2008 | 500 | Inpatients | US | Retrospective single-center | Yes | Yes | Yes | 70 | 97.8 | - | 26.2 | 43 | 34.2 | 49.6 | 42.2 | 10.8  (30-day) | _ | _ | _ | 5.8 |
| Corrales-Medina et al [34] | 2009 | 206 | Inpatients ^d^ | US | Retrospective single-center | Yes | Yes | Yes | 68 | >95 | - | 19.2 | 33 | 24.3 | 48.5 | 55.3 | 12.6  (30-day) | _ | _ | _ | 10.7 ^h^ |

**Table S1.** Details of the setting, design and population of studies of cardiac complications in patients with community acquired pneumonia

^a^ Congestive heart failure, atrial fibrillation, severe angina or myocardial infarction or stroke [13]; acute coronary or ventricular insufficiency [17]; cardiovascular complications likely to necessitate continued hospitalization [22]; cardiac complications without further specification [23]; acute coronary syndrome and/or heart failure [28]; myocardial infarction, angina pectoris, revascularization by angioplasty/CABG or death secondary to coronary heart disease, cerebrovascular accident, congestive heart insufficiency or claudication [29]; myocardial infarction, atrial fibrillation or ventricular tachycardia or incident heart failure [30]; and myocardial infarction, atrial fibrillation, congestive heart failure or stroke [31]

^b^  Incident atrial fibrillation [10,14,15,31,32];cardiac dysrrhythmias/arrhythmias [16,26]; incident atrial arrhythmia [19], atrial flutter or fibrillation, and ventricular tachycardia, but excluding terminal arrhythmias [30]

^c^ Myocardial infarction [15,19,20,30,31,33]; Unstable angina [27]; acute coronary syndrome [32,34]

^d^ Pneumococcal pneumonia [11,20,30]; pneumococcal and H. influenzae pneumonia [34]

^e^ Median

^f^Inpatients without severe vital signs or metabolic abnormalities, altered mental status, suppurative complications or coexisting medical conditions requiring hospitalization [15]; inpatients who survived the first 48h of hospitalization [24], inpatients not initially admitted to the intensive care unit [25,28]; inpatients in Pneumonia Severity Index (PSI) risk classes I-II [27]

^g^ Inpatients admitted to the intensive care unit (ICU)

^h^ For acute coronary syndromes, patients from Musher et al (2007) [30] were included in Corrales-Medina et al (2009) [34]
