# Supplementary material for: Cardiac Complications in Patients with Community-Acquired Pneumonia: A Systematic Review and Meta-Analysis of Observational Studies
Source: PLoS Med. 2011 Jun 28;8(6):e1001048. doi: 10.1371/journal.pmed.1001048 (PMC3125176; doi:10.1371/journal.pmed.1001048)
Supplement: Table S2 — Definitions of cardiac complications used in studies of CAP. (DOC) [file pmed.1001048.s003.doc]

**Table S2.** Definitions of cardiac complications used in studies of community acquired pneumonia

| **Reference** | **Outcome** | **Definition** |
| --- | --- | --- |
| Fine et al [19] | Incident heart failure  Myocardial infarction  Incident cardiac arrhythmia | Development of new or worsening pulmonary edema or acute congestive heart failure documented clinically (rales, increased jugular venous pressure, S3 gallop, edema), associated with a chest x-ray appearance of pulmonary edema, cardiomegaly, vascular congestion, or congestive heart failure  Ischemia and damage to the heart muscle will be documented by two of three criteria: chest pain, electrocardiographic changes, or positive cardiac enzymes (CPK MB). A subendocardial myocardial infarction is defined as ST segment and T wave changes without the formation of Q waves, while a transmural myocardial infarction has new Q waves or a clear loss of R waves  Development of newly recognized or worsened chronic atrial fibrillation, atrial flutter, supraventricular tachycardia, or multifocal atrial tachycardia documented in progress notes, or by electrocardiogram, Holter report, rhythm strips or other forms of electrocardiographic monitoring |
| Fine et al [22] | Incident heart failure  Myocardial infarction  Incident cardiac arrhythmia | Physician documentation in progress notes of new onset or worsening of chronic congestive heart failure, pulmonary congestion, or pulmonary edema treated with intravenous diuretics  Physician documentation in progress notes of acute myocardial infarction  Physician documentation in progress notes of new onset or worsening of chronic arrhythmias (including but not limited to atrial fibrillation , atrial flutter, paroxysmal atrial tachycardia, multi-focal atrial tachycardia, supraventricular tachycardia,  ventricular tachycardia, ventricular fibrillation, or ventricular bigeminy, trigeminy, quadrigeminy), resulting in systolic blood pressure <90 mm Hg, chest pain, or syncope |
| Musher et al [30] | Incident heart failure  Myocardial infarction  Incident cardiac arrhythmia | Composite of physical findings, laboratory findings (such as increases in B-natriuretic peptide of >500 pcg/mL), chest radiograph (for new or increased pulmonary vascular congestion), and echocardiograms consistent with new or worsening heart failure  New electrocardiographic abnormalities (i.e., ST segment elevation or depression or Q waves) accompanied by troponin I serum levels of 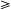0.5 ng/mL  Electrocardiographic evidence ofo atrial flutter, atrial fibrillation, or ventricular tachycardia, but excluding terminal arrhythmias |
| Becker et al [31] | Incident heart failure  Myocardial infarction  Incident atrial fibrillation | Radiology report describing compatible changes or diagnosis documented clinically by housestaff in progress notes  Discharge diagnosis of myocardial infarction, documentation of an myocardial infarction by a physician in the progress notes, or evidence of at least two of the following criteria: (a) cardiac chest pain; (b) positive cardiac enzymes or (c) a positive ECG  Evidence of this diagnosis on a rhythm strip or electrocardiogram |
| Ramirez et al [33] | Myocardial infarction | Typical increase and gradual decrease of biochemical markers of myocardial necrosis and at least 1 of the following: ischemic symptoms, development of pathologic Q waves on electrocardiogram, electrocardiogram changes indicative of ischemia (ST segment elevation or depression), or coronary artery intervention (e.g., coronary angioplasty); or (2) pathologic findings of acute myocardial infarction. Patients with concomitant diagnosis of severe sepsis were excluded from the group of patients with acute myocardial infarction |
| Corrales-Medina et al [34] | Acute coronary syndrome | ACS was considered if any of the following criteria were met: a) presence of evolving diagnostic changes in electrocardiogram or diagnostic cardiac biomarkers; or b) electrocardiogram findings consistent with ischemia plus cardiac symptoms or signs. Cases were then reviewed by a senior cardiologist pneumonia status of the patient |
