# Supplementary material for: Cardiac Complications in Patients with Community-Acquired Pneumonia: A Systematic Review and Meta-Analysis of Observational Studies
Source: PLoS Med. 2011 Jun 28;8(6):e1001048. doi: 10.1371/journal.pmed.1001048 (PMC3125176; doi:10.1371/journal.pmed.1001048)
Supplement: Text S2 — Search strategy. (DOC) [file pmed.1001048.s005.doc]

**Text S1. Search strategy**

Database: Ovid MEDLINE(R) In-Process & Other Non-Indexed Citations and Ovid MEDLINE(R) <1950 to Present>

--------------------------------------------------------------------------------

1 Community-Acquired Infections/

2 community acquired.tw.

3 1 or 2

4 exp Pneumonia/

5 (pneumon$ or pneumococcus).tw.

6 5 or 4

7 3 and 6

8 exp treatment outcome/

9 Prognosis/

10 "Length of Stay"/

11 Morbidity/

12 Mortality/ or "hospital mortality"/

13 Patient Readmission/

14 Survival/

15 exp cardiovascular Diseases/

16 exp stroke/

17 exp Brain Ischemia/

18 exp "embolism and thrombosis"/

19 survival analysis/

20 disease-free survival/

21 (cardiovascular or cardiac or heart or vascular or myocardial infarction$ or hypovolemia or pericarditis or arrhythmia$ or shock$ or coronary).tw.

22 (stroke or cerebral vascular accident$ or cerebrovascular accident$ or brain ischem$ or cva or ischem$ attack$ or tia).tw.

23 ((pulmonary or lung) adj2 embol$).tw.

24 (mortality or morbidity or readmission$ or complication$ or death$ or rehospitali$ or survival).ti.

25 or/8-24

26 7 and 25

27 remove duplicates from 26

28 animals/ not humans/

29 27 not 28
